# ChatGPT and Gemini for Patient Education: A Comparative Analysis of Common Pediatric Exanthematous Conditions

**DOI:** 10.7759/cureus.82705

**Published:** 2025-04-21

**Authors:** Amrutha Reshi, Nikhil Arora, Tanupriya Singh

**Affiliations:** 1 Psychiatry, Bhagawan Sri Balagangadharanatha Swamiji (BGS) Global Institute of Medical Sciences, Bengaluru, IND; 2 Pediatrics, Government Medical College, Patiala, IND; 3 Pediatrics, Guru Gobind Singh Medical College and Hospital, Faridkot, IND; 4 Pediatric Medicine, The Doctor's Hub Polyclinic, Dubai, ARE

**Keywords:** ai tools, artificial intelligence (ai), chatgpt, google gemini, patient education, pediatric, skin conditions

## Abstract

Introduction: Varicella, hand, foot, and mouth disease (HFMD), and measles are some of the common causes of fever with rash in the pediatric age group. ChatGPT and Gemini are effective large language models (LLMs) for parents to understand their child’s condition. Therefore, considering the growing popularity of artificial intelligence (AI), LLMs, and their ability to disseminate health information, assessing ChatGPT's (OpenAI, San Francisco, CA, USA) and Gemini's (Google LLC, Mountain View, CA, USA) quality and accuracy is essential.

Materials and methods: A cross-sectional study was conducted on responses generated using AI for common causes of fever with rash in the pediatric age group, namely varicella, HFMD, and measles. ChatGPT and Gemini were used for the generation of brochures for patient education. The responses generated were evaluated using the Flesch-Kincaid Calculator (Good Calculators: https://goodcalculators.com/), the QuillBot plagiarism tool (QuillBot, Chicago, IL, USA), and the modified DISCERN score. Statistical analysis was done using R version 4.3.2 (R Foundation for Statistical Computing, Vienna, Austria, https://www.R-project.org/), and unpaired t-tests were used to compare the various scores. A p-value of less than 0.05 was considered statistically significant.

Results: It was found that ChatGPT generates a higher word count as compared to Gemini (p=0.047). Sentences, average words per sentence, average syllables per word, ease score, and grade level between the two AI tools were statistically insignificant (p>0.05). The mean reliability score was 3/5 in the case of Gemini versus 2.67/5 in ChatGPT, but the difference was statistically insignificant (p=0.725).

Conclusions: This study highlights that ChatGPT generates more word count than Gemini, and the finding was statistically significant (p=0.047). Additionally, there is no significant difference in the average ease score or grade score for common pediatric exanthematous conditions: varicella, HFMD, and measles. Future research should focus on improving AI-generated health content by incorporating real-time validation mechanisms, expert reviews, and structured patient feedback.

## Introduction

Fever with rash is a common problem in the pediatric age group. It can range from minor viral illness or any drug allergy to serious life-threatening causes such as dengue hemorrhagic fever [1]. Some common causes are varicella, hand, foot, and mouth disease (HFMD), and measles [1]. Educating parents about pediatric exanthematous diseases is essential for early recognition, timely management, and reducing transmission risks.

Various artificial intelligence (AI) tools are being utilized by the public for various uses, including searching for information about various health conditions. One such tool is ChatGPT (OpenAI, San Francisco, CA, USA), based on a natural language processing model that is being widely used [2]. It had gained 100 million users in its initial two months of launch in 2022 [3]. Later in 2023, Gemini was released (Google LLC, Mountain View, CA, USA), a large language model like that of ChatGPT [4]. Now it is one of the primary competitors of ChatGPT [4].

The conventional search engines find up-to-date information from the web by providing various links to relevant pages. Still, in contrast, these AI tools generate responses based on their trained knowledge and real-time web content, offer direct answers, and engage in human-like conversation. These AI models are easily accessible, convenient, and fast. While search engines provide information on diseases with references to sources, some AI tools' responses lack direct references, posing challenges to the verification of medical accuracy.

These language-based models can be an effective tool for the parents to understand their child’s condition and become more health-literate [5]. Therefore, evaluating the readability, reliability, and accuracy of ChatGPT and Gemini in generating pediatric health information is essential.

This study aimed to evaluate the accuracy, dependability, and readability of responses from ChatGPT and Gemini to frequently asked questions on varicella, HFMD, and measles and compare their effectiveness in creating patient education guides based on clarity and ease of understanding.

## Materials and methods

A cross-sectional study was conducted from August 6th to August 12th, 2024. First, the responses were generated using AI for three common diseases in pediatrics, as selected, namely varicella, HFMD, and measles. Two AI tools were selected, namely ChatGPT 3.5 and Gemini, for the generation of brochures for patient education [6,7]. As there were no human participants, the study was deemed exempt. However, the study adheres to principles of ethical AI evaluation in medical research.

Each AI tool was provided with identical standardized prompts to generate patient education guides for varicella, HFMD, and measles. The chatbots were given one prompt after the other to write a patient education guide for three diseases. The prompts given were as follows: “Write a patient education guide for 'varicella,'" “Write a patient education guide for 'hand-foot-mouth disease,'" and “Write a patient education guide for 'measles.'" The responses generated were then collected in a Microsoft Word document (Microsoft Corporation, Redmond, WA, USA) for further analysis.

All responses were further graded using the Flesch-Kincaid Calculator (Good Calculators: https://goodcalculators.com/), which assessed the readability, ease of understanding, and grade level required for comprehension [8]. The QuillBot plagiarism tool (QuillBot, Chicago, IL, USA) was used to calculate a similarity percentage to ensure the originality of the content produced by the chatbots [9]. Lastly, the reliability of AI-generated content was evaluated using the modified DISCERN score, which assesses conciseness, reliability, balance, references, and uncertainty on a 0-5 scale [10]. Each criterion is scored, and the total score provides an overall measure of the quality of the information, with higher scores indicating greater reliability of the information.

Data was analyzed using R version 4.3.2 (R Foundation for Statistical Computing, Vienna, Austria, https://www.R-project.org/), with comparisons between ChatGPT and Gemini performed using an unpaired t-test for numerical variables [11]. A p-value of less than 0.05 was considered significant. The correlation between ease and reliability scores was compared using Pearson’s correlation coefficient. Welch's two-sample t-test was used to compare the means between ChatGPT and Gemini. The normality of the variables was assessed using the Shapiro-Wilk Test. The equality of variances of the two groups was assessed using Levene’s test.

## Results

Based on the p-values obtained in Table 1, there is a statistically significant difference between words generated by the two AI tools (p=0.047). Sentences, average words per sentence, and average syllables per word between the two AI tools were statistically insignificant.

**Table 1:** Characteristics of responses generated by ChatGPT and Gemini * t-test; p-values <0.05 are considered statistically significant. SD: standard deviation

Variables	ChatGPT		Gemini		p-value*
	Mean	SD	Mean	SD	
Words	655.67	109.04	381.33	14.64	0.047
Sentences	61.67	9.71	49.00	8.54	0.165
Average words per sentence	10.60	0.27	7.97	1.62	0.050
Average syllables per word	1.77	0.06	1.80	0.17	0.768
Grade level	9.40	0.70	8.77	1.55	0.555
Ease score	46.63	5.00	46.47	13.43	0.985
Similarity percentage	47.27	10.07	22.07	21.08	0.135
Reliability score	2.67	1.15	3.00	1.00	0.725

**Figure 1:**
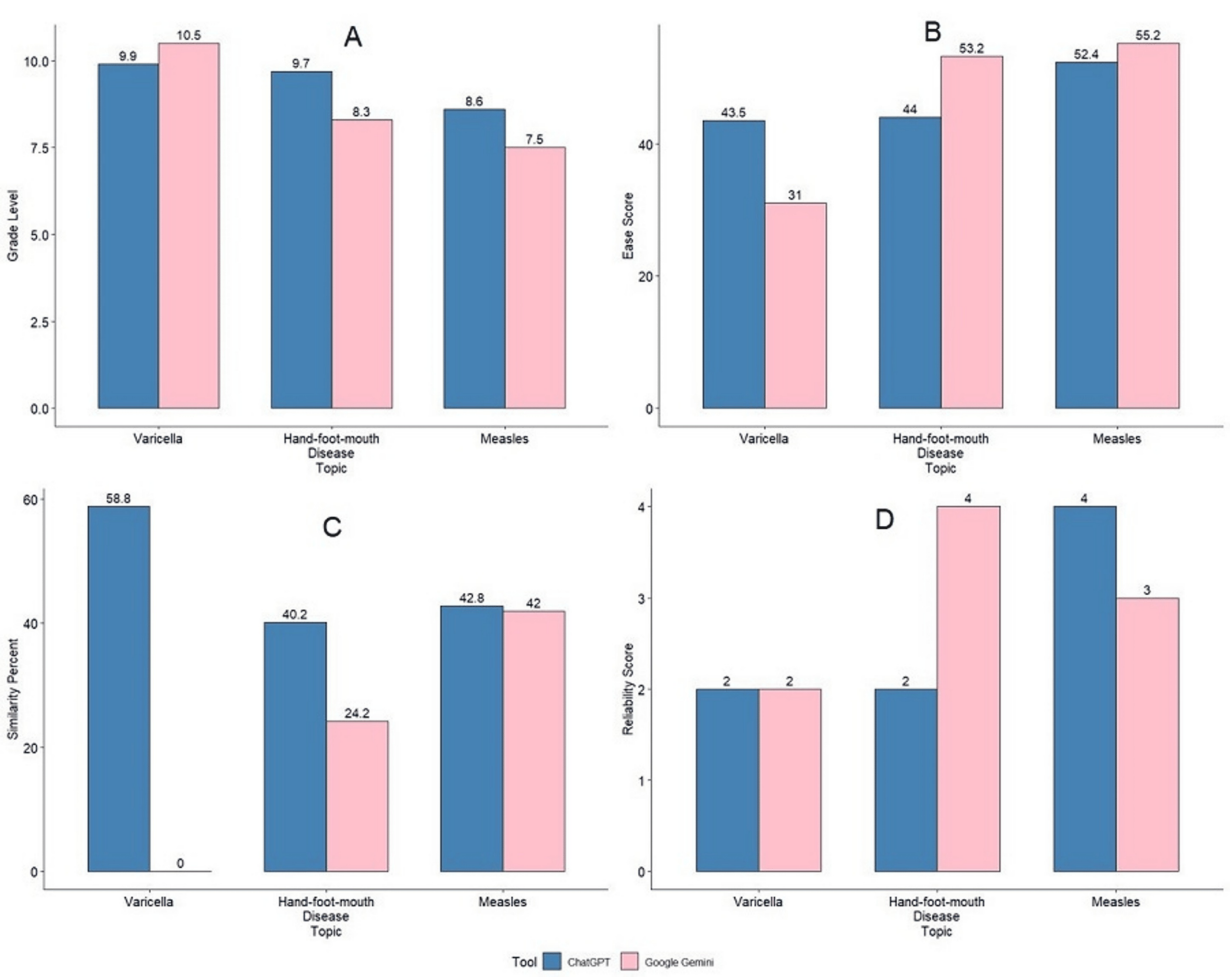
Graphical representation of the comparison between grade level, ease score, similarity percent, and reliability score for the patient education guide generated by ChatGPT and Gemini

The mean grade level for the three diseases was found to be 9.40 for ChatGPT versus 8.77 for Gemini. The difference was statistically insignificant (p=0.555). The mean ease score seen for ChatGPT and Gemini was 46.63 and 46.47, respectively (p=0.985). According to the modified DISCERN score, the mean reliability score was 3/5 in Gemini's case, whereas in ChatGPT's case, it was 2.67/5, but the difference was statistically insignificant (p=0.725). Figure 1 shows the graphical representation.

## Discussion

This cross-sectional study compares responses from two AI tools, ChatGPT and Gemini, for brochures on patient education for varicella, measles, and HFMDs. This study revealed that ChatGPT generates more word count than Gemini, and the finding was found to be statistically significant (p=0.047). The mean reliability score according to the modified DISCERN score was 3/5 for Gemini, whereas in the case of ChatGPT, it was 2.67/5, but the difference was statistically not significant.

The recent popularity of AI tools has potential for broad application in the field of medicine [12]. To support this, Božić stated that AI is revolutionizing personalized medicine by tailoring treatment plans to individual patient characteristics, enhancing efficacy, and reducing side effects. Additionally, AI-driven virtual assistants and chatbots can potentially enhance patient engagement by providing instant, personalized health information, improving access to healthcare resources, and promoting health literacy [12]. Furthermore, Hernandez et al. demonstrated that 98.5% of the responses regarding type 2 diabetes education, delivered by ChatGPT, were deemed appropriate, proving that the AI model consistently delivered accurate information aligned with the standard of care for managing type 2 diabetes and its related complications [13].

In this study, the Flesch-Kincaid grade level is used to assess the grade level of the responses generated by ChatGPT and Gemini. The mean grade level for the three diseases was found to be 9.40 (0.70) for ChatGPT and 8.77 (1.55) for Gemini. A very similar finding was seen in a study by Rouhi et al., which revealed grade level to be 9.4 (2.0) for ChatGPT, indicating difficult readability at the ninth-grade reading level [14]. Subsequently, this study showed that the mean ease score seen for ChatGPT and Gemini was 46.63 (5.00) and 46.47 (13.43), respectively. A slightly lower value was noticed in a study by Behers et al., which showed mean ease scores of 31 and 36.4 for ChatGPT and Gemini, respectively [15]. However, both studies interpret that the study was suitable for people at the college level. A statistically significant finding in this study was that the mean words generated by ChatGPT was 655.67 (109.04) and by Gemini was 381.33 (14.64). Similarly, a study by Yalla et al. showed that the words generated by AI tools ChatGPT and Bing were 222.26 (29.17) and 100.77 (38.33), respectively, whose comparison led to a significant statistical value of p<0.0001 [16]. A higher word count could indicate more in-depth information being provided by the chatbot, which could further enhance the reliability and quality of information being generated [15,16].

The concern for plagiarism also arises with the increasing usage of AI tools such as ChatGPT and Gemini. AI tools use preexisting literature and hence can generate sentences mimicking original work. When major plagiarism is detected, published work can severely damage the reputations of the plagiarist, coauthors, reviewers, editors, and their institutions. Retraction, the withdrawal of an article due to research misconduct like plagiarism, has increased significantly in recent decades and can permanently harm an author's reputation. Salvagno et al. noted that it's common for people to unintentionally replicate the ideas, statements, or written content of others, which can lead to plagiarism when proper attribution is not provided. AI tools like ChatGPT may fall into this form of plagiarism, but they can also be designed to paraphrase content like human writers do. Nevertheless, relying on such tools solely to reword existing texts in order to lower plagiarism detection by rewriting another author's work using different language would not be considered an acceptable practice in scientific research [17].

The DISCERN score is a tool used to assess the quality of written health information, particularly patient information. Higher DISCERN scores indicate better quality information. In this study, the average DISCERN score was found to be 2.67 (1.15) and 3.00 (1.00) for ChatGPT and Gemini, respectively. A similar result was seen in a comparative study by Hancı et al., which showed that ChatGPT had a reliability score of 2.29 ± 0.91 and Gemini scored 2.37 ± 0.51. Furthermore, the comparison between the two was statistically significant (p<0.001) [18]. However, a study done by Dursun et al. showed a significantly higher DISCERN score of 19.7 ± 2.15 in ChatGPT and 21.1 ± 2.63 in Gemini [19]. There was also no correlation between the ease score and reliability score between the two software, indicating that there is a possibility that although easier to read and understand, the information could still be unreliable. However, a more in-depth analysis is needed.

Limitations

This study has some notable limitations, the most important one being the comparison between only two of the AI tools, ChatGPT and Gemini, at the time they were more commonly used and researched; however, with other AI chatbots being introduced into the market, it is essential to further compare and analyze the quality of information being produced by all models. It is also essential for future studies to investigate the accuracy of the information provided; this study only assessed the reliability, and an in-depth analysis of accuracy would be extremely beneficial.

The chatbots were only given the prompts once to ensure consistency, and this does not account for the variance of responses generated by the models, a feature future studies can look into. Additionally, this study demonstrated the trends related to pediatric exanthematous conditions only. Hence, this study has restricted information on patient education responses generated by AI tools on other diseases. Another significant limitation is that the present study utilized ChatGPT 3.5 and may not produce the most up-to-date information. Finally, with new and pivotal medical updates every day, the promise of AI tools to keep up with the expansive information and provide suitable education to patients is questionable.

## Conclusions

ChatGPT generated significantly longer responses than Gemini, but there was no significant difference in readability, ease of understanding, or reliability. Additionally, there is no significant difference in the average ease score or grade score for common pediatric exanthematous conditions, varicella, HFMD, and measles. There is no correlation between the ease and reliability scores between the two software.

Further studies must focus on ensuring accuracy and reliability with up-to-date, evidence-based content. The content generated should be verified to ensure the language's personalization and simplification to enhance readability and include interactive features and visual aids, ensuring cultural sensitivity and accessibility, with options for different languages and formats. More validation tools for AI-generated medical content and investigating AI models across different languages and literacy levels should also be done.

## References

[1] Muzumdar S, Rothe MJ, Grant-Kels JM (2019). The rash with maculopapules and fever in children. Clin Dermatol.

[2] (2024). OpenAI. https://openai.com/product.

[3] Milmo D. ChatGPT reaches 100 million users two months after launch (2024). ChatGPT reaches 100 million users two months after launch. The guardian [Internet.

[4] Dupré MH (2024). OpenAI rages at report that Google’s new AI crushes GPT-4. https://futurism.com/the-byte/openai-report-google-ai-gpt-4?utm_medium=email&utm_source=transaction..

[5] Wang X, Sanders HM, Liu Y (2023). ChatGPT: promise and challenges for deployment in low- and middle-income countries. Lancet Reg Health West Pac.

[6] (2024). ChatGPT. https://chat.openai.com.

[7] (2024). Gemini. https://gemini.google.com/.

[8] (2024). Flesch-Kincaid calculator. https://goodcalculators.com/flesch-kincaid-calculator/.

[9] Xuyen NT (2023). Using the online paraphrasing tool Quillbot to assist students in paraphrasing the source information: English-majored students’ perceptions. Proceedings of the 5th Conference on Language Teaching and Learning 2023.

[10] Uzun O (2023). Assessment of reliability and quality of videos on medial epicondylitis shared on YouTube. Cureus.

[11] (2024). The R Project for statistical computing. https://www.r-project.org/.

[12] Boži̇ć V (2024). The role of artificial intelligence in increasing the health literacy of patients. Int J Digit Health Patient Care.

[13] Hernandez CA, Vazquez Gonzalez AE, Polianovskaia A (2023). The future of patient education: AI-driven guide for type 2 diabetes. Cureus.

[14] Rouhi AD, Ghanem YK, Yolchieva L (2024). Can artificial intelligence improve the readability of patient education materials on aortic stenosis? A pilot study. Cardiol Ther.

[15] Behers BJ, Vargas IA, Behers BM, Rosario MA, Wojtas CN, Deevers AC, Hamad KM (2024). Assessing the readability of patient education materials on cardiac catheterization from artificial intelligence chatbots: an observational cross-sectional study. Cureus.

[16] Yalla GR, Hyman N, Hock LE, Zhang Q, Shukla AG, Kolomeyer NN (2024). Performance of artificial intelligence chatbots on glaucoma questions adapted from patient brochures. Cureus.

[17] Salvagno M, Taccone FS, Gerli AG (2023). Can artificial intelligence help for scientific writing?. Crit Care.

[18] Hancı V, Ergün B, Gül Ş, Uzun Ö, Erdemir İ, Hancı FB (2024). Assessment of readability, reliability, and quality of ChatGPT®, BARD®, Gemini®, Copilot®, Perplexity® responses on palliative care. Medicine (Baltimore).

[19] Dursun D, Bilici Geçer R (2024). Can artificial intelligence models serve as patient information consultants in orthodontics?. BMC Med Inform Decis Mak.

